# The time sensitivity of aspirational interventions: Evidence from a role-modeling RCT

**DOI:** 10.1371/journal.pone.0350832

**Published:** 2026-06-15

**Authors:** Prateek Chandra Bhan, Max Schroeder, Damiano Turchet, Jinglin Wen

**Affiliations:** 1 Cluster of Excellence Politics of Inequality and Department of Economics, University of Konstanz, Konstanz, Germany; 2 Durham University Business School, Durham University, Durham, United Kingdom; 3 Department of Economics, University of Warwick, Coventry, United Kingdom; 4 Department of Economics, University of York, York, United Kingdom; University College London, UNITED KINGDOM OF GREAT BRITAIN AND NORTHERN IRELAND

## Abstract

This paper investigates the short-run effects of an aspiration-raising intervention delivered as part of a randomized controlled trial among postgraduate students at a UK university during the Covid-19 pandemic. We document suggestive evidence, that a video-based role-modeling intervention led to an immediate increase in aspirations and a delayed increase in self-reported effort. However, both effects dissipated within a few weeks. Our findings suggest that timing and reinforcement are important considerations for the sustained effectiveness of aspiration-building strategies.

## 1 Introduction

Ray [1], suggests that aspirations can motivate individuals towards better outcomes by promoting effort and perseverance (see also [2]). Further, recent studies ([3–5]) showed that relatively cost-effective interventions can help increase aspiration levels in a variety of settings.

In this paper, we contribute to the growing literature on aspiration and effort, by studying the aspirations and effort dynamics in a group of postgraduate students in a university in Scotland. We track the students’ aspirations and effort levels over the sensitive period of the 2020-fall semester. We design and implement a cost-effective role-modeling intervention to raise students’ aspirations. In line with the empirical literature ([6–8]), we find suggestive evidence that the intervention raises aspirations relative to the control group, but that the effect quickly disappears after a couple of weeks. Further, and in line with the theoretical literature ([2]), we find suggestive evidence that the intervention raises effort in subsequent periods, but this effect is also short-lived.

Our results motivate an important direction for further research. While our findings reconfirm the theoretical link between aspirations and effort, the rapid fading out of the effects suggest an important role for the timing of aspiration-increasing intervention studies. If both aspirations and effort effects are short-lived, one-off interventions may at times be insufficient to produce lasting positive outcomes, unless timed carefully.

Limited research has investigated the time-sensitivity of aspiration-building interventions, and we believe that this approach can shed some light on the mixed empirical evidence on the sustainability of role modeling treatments ([8,9]). As such our paper provides an important direction for future research and policy.

The paper is structured as follows: in Section 2 we describe the experiment and present the results, in Section 3 we develop a simple model of aspiration and effort determination, Section 4 provides a further discussion on the results and draws some concluding remarks.

## 2 Experiment

### 2.1 Background

We invited postgraduate taught students (PGT) from the College of Social Sciences (CoSS) at the University of Glasgow to participate in an RCT by email. Ethical approval for this study was obtained from the Ethical Review Committee of the University of Glasgow (reference number 400190225; approved on 23 September 2020). All participants provided informed consent by signing the consent form at the start of the survey before answering any study questions. The study was also pre-registered with the AEA RCT Registry (AEARCTR-0006520).

**Fig 1:**
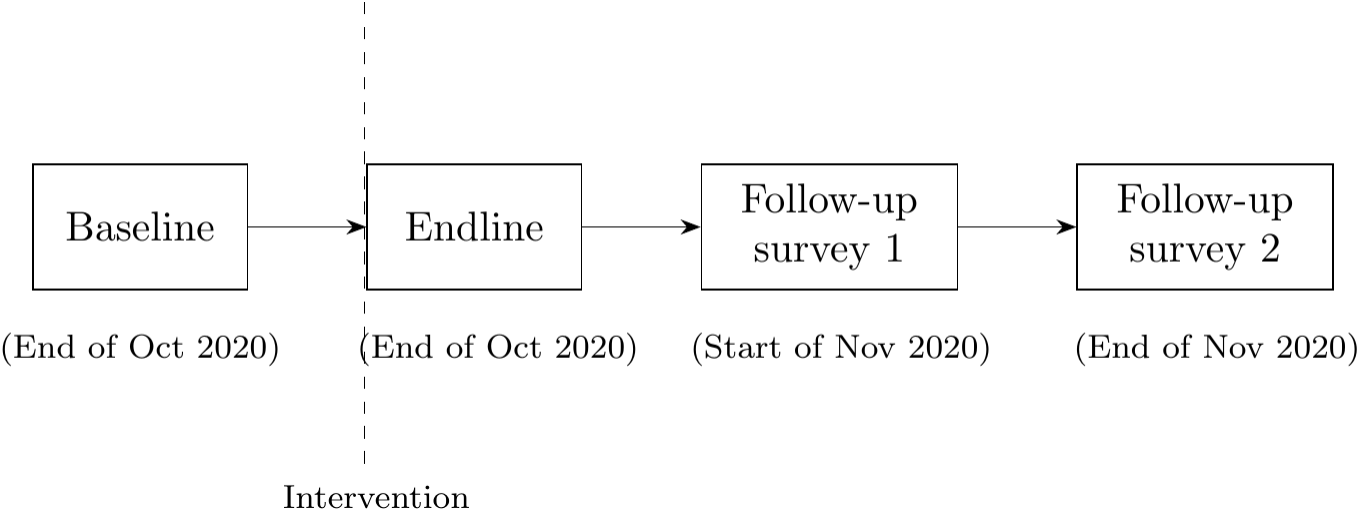
Project timeline.

The initial stage of the experiment consisted of a baseline survey questionnaire, followed by a Zoom webinar session, immediately followed by an endline survey. Later on, we contacted these students via email to complete two subsequent online surveys. Students were strategically invited at the start of their course to ensure that the timeline and follow-up waves of surveys tracked their aspirations through the autumn term of 2020 (September – December 2020, see Fig 1). This allowed us to observe how students’ aspirations developed over the course of an academic semester. Students begin their studies in September and sit a number of exams over the Christmas exam period, which provides a natural goal-point on which to focus aspirations. An incentive scheme was put in place to reimburse the students’ time and to encourage participation. The incentive plan, invitation emails, survey questions (At each round of survey, participants gave consent by signing the form at the start, before answering the relevant questions. For further details, see the questionnaire from the link in S2 File.) and scripts of the Zoom webinar are available in S2 File. Further details on the construction of outcome variables, including the exact wording of survey questions, the composition and scoring of indices, normalization procedures, reliability statistics, are provided in S3 File. The initial participant pool consisted of 600 students that started filling the initial online-survey. However, over half of the students did not complete the survey, or did not consent to participate in the RCT. Due to this, the baseline sample consisted of 246 students who completed the survey and agreed to participate in the RCT. These were randomised into three groups. At the time of the intervention, we had a further fall in the number of participants leaving an effective sample of 121 in the subsequent rounds. The number of students in each phase of the data collection is illustrated in Fig 1 in S1.

The survey and experiment were conducted throughout one of the high phases of the Covid-19 pandemic, which likely contributed to the high attrition rate. However, we find no evidence of differential attrition based on treatment status, as both raw and covariate adjusted attrition rates are similar for treatment and control groups. Details are shown in Table 3 in the S1 File.

Table 1 shows the baseline characteristics for all student who participated in the experimental sessions. Table 2 in S1 File shows the balancing tests for all students who completed the baseline survey (Participants were randomised into treatment and control groups following completion of the baseline survey. They were not made aware of which group they were assigned to and received links to Zoom sessions via email, making it unlikely that the large drop in participation between baseline and endline was related to differential treatment assignment. Indeed Table 2 in S1 File shows that baseline characteristics are balanced across treatment and control groups for the full baseline sample. Additionally, Table 4 is S1 File compares students in the linked analysis sample with other baseline respondents. The linked sample is broadly similar on most baseline observables, although it contains a somewhat higher share of white students and a somewhat lower share of female students.). Our final, linked sample skews heavily male (∼80%), with an average age between 25 and 27 years. Nearly 40% of students are from non-white ethnicity backgrounds. The composition of our sample reflects the underlying structure of postgraduate taught programmes in the College of Social Sciences at the University of Glasgow. In particular, these programmes are heavily weighted towards subjects such as management, finance, and accounting, which tend to enroll a high proportion of male and international students. As a result, our sample skews towards male and non-UK students, but this largely mirrors the population from which participants were drawn rather than arising from differential selection into the study.

**Table 1:** Balancing checks.

	Treatment			Control			
Variable	N	Mean	S.D.	N	Mean	S.D.	p-value
Age (years)	77	26.64	6.81	42	26.88	5.34	0.841
Female (1-female, 0-male)	77	0.21	0.41	42	0.24	0.43	0.705
White (1-white, 0-others)	77	0.60	0.49	42	0.55	0.50	0.603
Education PGDE (1-yes, 0-other programmes)	77	0.10	0.31	42	0.14	0.35	0.532
Aspiration (s.d.)	77	−0.09	1.00	42	0.17	0.99	0.166
Educational Aspiration (s.d.)	77	−0.03	0.98	41	0.06	1.04	0.658
Hope (s.d.)	77	−0.04	1.00	42	0.08	1.01	0.534
Confidence (s.d.)	77	−0.07	0.99	42	0.13	1.01	0.291
Effort (1-no missed lectures, 0-missed any)	76	0.83	0.38	41	0.83	0.38	0.997
Effort (1-no missed tutorials, 0-missed any)	76	0.91	0.29	41	0.93	0.26	0.729

The table reports group means and standard deviations for the linked analysis sample. The final column reports the exact p-value from a two-sample test of equality of means between the treatment and placebo groups. Standardized outcomes are scaled to mean 0 and standard deviation 1.

### 2.2 Design

Students were randomised into three groups that determined their treatment assignment. Treatment 1 and 2 contained videos intended to boost students’ aspirations but differed in intensity. The video shown to treatment group 2 was 35 minutes long, while the video shown to treatment group 1 contained an additional 5 minutes. A 35-minute-long placebo video was shown to the control group. The intervention videos are available in S2 File. A production team was hired to script and edit these videos using audio-visual data that was available on the internet. This further adds to the cost-effectiveness of the intervention: inspiring content is readily available to be harnessed into a tool for development.

The intervention videos were designed to influence effort by presenting narratives of perseverance, resilience, and goal-directed behaviour through high-achieving role models across athletics, business, and academia. By showcasing both the challenges and strategies of these individuals, the videos aim to foster aspirational updating, whereby participants adjust their perception of what they can achieve through effort, and to highlight the tangible rewards of persistence, potentially increasing perceived returns to effort. This approach builds on prior research in psychology and behavioral science showing that exposure to relatable, successful role models can shape motivation and goal-directed behaviour [5,10]. The design closely follows established experimental protocols by presenting carefully curated, real-world examples of success across multiple domains, ensuring both theoretical coherence and external validity in contexts where participants encounter realistic and relatable role models ([1]). The role models were selected to span a diverse set of characteristics and professional trajectories, including differences in gender, ethnicity, and field of achievement (i.e., academia, business and sport). This design aims to increase the likelihood that participants could identify with at least some of the featured individuals, thereby strengthening the credibility of the aspirational signal.

The placebo group was shown an edited video of similar duration, which showcased cultural and historical aspects of the city of Glasgow and the surrounding countryside, portrayed through tourist lenses (This content was in style and content similar to traditional documentaries or travel video-blogs that were readily available via traditional or social media channels.). The video was chosen to match the intervention in terms of attention, visual engagement, and overall format, while deliberately avoiding any references to effort, perseverance, or achievement. This ensures that any observed treatment effects can be attributed to the aspirational content of the intervention rather than differences in exposure time, engagement, or media format.

Participants in each group attended a Zoom webinar, where they were shown a video based on their treatment allocation. The intervention content and delivery was inspired from several role-modeling studies using multi-media channels to successfully influence human behaviour in general [11–13], and aspirations in particular [5,14,15]. During and before the session, we embedded numerous compliance checks in the form of mandating participants to keep their videos on, or tracking the views on the unlisted YouTube video used for the intervention, among others. These include recording the time participants took to fill out the online surveys. Further, students were notified that as a part of the webinar, some questions about the video would appear as part of the survey questionnaire, which they had to answer correctly to ensure authentic participation on their part. These questions ensured that students had an incentive to pay close attention to the videos. Lastly, the intervention videos were kept in an unlisted inactive format at all times except for the duration of the webinar. This prevented any unforeseen sharing or re-watching.

Information was collected on key variables of interest, such as aspirations and hope, using self-reported measures for academic and general aspirations [16,17] and the adult hope scale (AHS) ([18]), respectively. The general aspiration index and the adult hope scale are created by aggregating a number of survey item responses. Educational aspirations refer to the numerical grade (A1 to F) on a scale of 0–12. We also collected data on effort by asking the students about the number of lectures and tutorials they missed in the past week. For more details on the construction of the different outcome measures see S3 File.

Making a departure from our pre-analysis plan (PAP) (AEARCTR-0006520), we combine treatments 1 and 2 to evaluate the effect of ’any aspirational video’ on student effort and aspirations against the placebo. This is driven by concerns about a lack of sufficient power to detect treatment effects in our already small sample. The nature of the content does not differ markedly between the two treatment videos. While treatment 1 is slightly stronger due to an additional 5 minutes duration, its message is implicitly the same as treatment 2. As reported in the PAP, we could not neatly disentangle the key messages of hope between the two treatments and thereby resort to the above-mentioned approach of bunching the two treatments to attain a larger sample of treated students and comparing the outcomes of ’any treatment’ group to the placebo group students. Table 1 shows the sample balance between the treatment and placebo groups. Lack of power additionally leads us to change the baseline specification from a difference-in-difference design to an Ancova model. We include the DiD specification in Table 9 in S1 File. The Ancova specification is as follows:


$$\begin{array}{c} Y_{it}=\alpha +\beta_{1}D_{i}+Y_{i0}+\psi X_{i}^{\prime }+\epsilon_{i}, \end{array}$$ (1)

where *Y*_*it*_ denotes the outcomes of interest, *D*_*i*_ is an indicator equal to one if the student is in the treatment group (treatment 1 or 2), and 0 otherwise, *Y*_*i*0_ controls for the value of outcomes at baseline, ${\text{X}}_{i}$ is a vector of control variables and $\epsilon_{i}$ is the error term. Control variables include age, gender, race indicators, and a binary indicator for enrollment in the Professional Graduate Diploma in Education (PGDE) programme.

### 2.3 Experimental results

Table 2 presents the treatment effects assessed using equation (1). Panel (a) shows the effects on the psychological outcomes of aspiration, hope and confidence. We observe an increase of 0.29 standard deviations in the generalised aspiration index; however, the estimate is only significant at the 10% level and should be interpreted with caution given the marginal significance and sample size. The effect diminishes as we consider the 2-week and 4-week follow-up surveys. Although we also observe a strong and persistent increase in educational aspirations, the effect is not significant at any traditional level of statistical significance. In addition, we do not detect any significant effect on hope or confidence.

**Table 2:** Treatment effects (any treatment vs placebo).

Panel (a): Psychological Outcomes	Endline	Follow-up 1	Follow-up 2
	(immediate)	(2 weeks)	(4 weeks)
**Aspiration**	0.285*	0.067	0.026
	(0.169)	(0.201)	(0.203)
	[0.094]	[0.739]	[0.900]
Observations	118	98	98
**Educational Aspiration**	0.113	0.151	0.144
	(0.119)	(0.133)	(0.144)
	[0.344]	[0.260]	[0.320]
Observations	118	96	97
**Hope**	−0.034	0.002	0.061
	(0.119)	(0.143)	(0.160)
	[0.772]	[0.987]	[0.702]
Observations	118	98	98
**Confidence**	0.062	0.234	−0.092
	(0.173)	(0.160)	(0.178)
	[0.720]	[0.147]	[0.604]
Observations	118	98	98
*Panel (b): Effort Outcomes*	**Endline**	**Follow-up 1**	**Follow-up 2**
	(immediate)	(2 weeks)	(4 weeks)
Not missing lectures/tutorials	0.023	0.193*	0.106
	(0.085)	(0.115)	(0.105)
	[0.789]	[0.097]	[0.319]
Not missing lectures	−0.023	0.254**	0.048
	(0.083)	(0.108)	(0.098)
	[0.787]	[0.022]	[0.627]
Not missing tutorials	−0.005	−0.054	0.132
	(0.064)	(0.087)	(0.094)
	[0.939]	[0.533]	[0.162]
Observations	116	95	96

The table reports ANCOVA treatment effects controlling for baseline outcomes. Controls include the baseline value of the relevant outcome, baseline psychological outcomes, age, gender, an indicator for enrollment in the Education PGDE programme, and race indicators. Heteroskedasticity-robust standard errors are in parentheses, and exact p-values are reported in square brackets below. Significance levels: *** p < 0.01, ** p < 0.05, * p < 0.10.

In panel (b), we find an increase in effort during the 2-week follow-up survey. Since effort is measured retrospectively, the treatment cannot have a direct measurable effect on effort during the endline survey. In this light, our finding of null effects in the first column of panel b) serves as a reassuring falsification test of our effort measure (As a check on the validity of this self-reported measure, we note that there is no detectable effect of the intervention on effort immediately after the session, consistent with the expectation that any increase in effort would take time to materialize; this pattern provides an indirect falsification test, supporting the credibility of our measure despite potential reporting bias. We use the absence of immediate treatment effects on self-reported effort as a plausibility check: if participants had over-reported effort due to experimenter demand or social desirability, we might expect an effect immediately after the intervention. That we do not observe such an effect provides some reassurance that the measure captures genuine effort changes in the short run. Furthermore, it is unlikely that truth-telling or lying behaviour would be influenced by the intervention.). We find a 0.19 standard deviation increase in the probability of not having missed any lectures or tutorials in the previous week, although the estimate is modest in magnitude and subject to statistical uncertainty due to the small sample size. The magnitude of the effect approximately halves in the second follow-up and loses significance as a result. As seen in the subsequent rows, this effect is mainly driven by a reduction in missed lectures. Generally, we believe that attendance at lectures is a better proxy for students’ effort, as attendance at tutorials was rigorously monitored, providing an extrinsic motivation for students to attend.

In Table 5 in S1 File, we show that there are no significant differences for all psychological outcomes and efforts after treatment between treatment group 1 and 2. In Tables A6 and A7 in S1 File, we show the treatment effects by comparing treatment groups 1 and 2 to the placebo group. We find that the results on effort are consistent with those in Table 2 for both treatment groups. In Table 8 in S1 File, we repeat the same exercises as Table 2 but exclude controls in the specifications. Although the effect on aspiration becomes insignificant, we find the effect on effort holds in the first follow-up. We additionally report the results of a difference-in-difference specification in Table 9 in S1 File. These results are similar in magnitude and direction, but less precisely estimated. To assess sensitivity to attrition, we also report inverse-probability-weighted ANCOVA estimates in Table 10 in S1 File. These weighted estimates are qualitatively similar to the main results, although the immediate aspiration effect weakens, while the follow-up lecture-attendance effect remains the most robust effort result. We further conduct some limited heterogeneity analysis by baseline characteristics. While we find no interpretable patterns of heterogeneity with respect to gender, race or PGDE status, there seems to be some evidence that the intervention is less effective in raising aspirations for individuals with above median baseline aspirations, as indicated in Table 11 in S1 File.

Because the analysis considers multiple outcomes across survey waves, we report multiple-hypothesis adjustments in Table 12 in S1 File. The table shows the raw p-values alongside Benjamini-Hochberg q-values and Romano-Wolf stepdown-adjusted p-values, computed separately within outcome family and wave. As expected given the modest sample size, the adjusted results are weaker than the unadjusted estimates. In particular, the immediate aspiration effect does not survive either adjustment, while the strongest remaining signal is the increase in lecture attendance at the two-week follow-up, which remains marginally significant after correction. We therefore interpret the pattern of results as suggestive evidence of short-run effects rather than conclusive evidence of robust treatment impacts across outcomes.

Overall, while not strongly significant, our results are consistent with some of the empirical and theoretical findings in the literature. First, we provide evidence aligning with the literature finding that role modeling interventions can increase self-reported psychological measures, like aspirations ([3,5]). Second, the effect of role-modeling intervention is short-lived and the effect effectively disappears after 2 weeks ([19]). Thirdly, we note a delayed response in the level of effort exerted by the treatment group, which is in line with the theoretical framework developed by Dalton et al. [2]. More broadly, the results should be interpreted with caution given the modest sample size, attrition, and the use of self-reported measures of effort.

## 3 Model

To better understand the mechanism by which aspirations translate into effort choices, we sketch a simple conceptual model, that builds on the behavioural framework of Dalton et al. [2]. In line with their work, we assume that students are behavioural decision makers, that take their aspirations *g*_*t*_ as given, and make choices over exerting effort *e*_*t*_. The agent values effort at $ln(e_{t})$, but effort is psychologically costly. Furthermore, the agent pays a psychological cost for not living up to their own aspiration levels, which is modeled as a quadratic cost for deviating from their aspiration level (*g*_*t*_ − *e*_*t*_)^2^.

The model can be summarised as the agent solving the following maximisation problem:


$$\begin{array}{c} e_{t}^{*}(g_{t})=\text{arg}\max_{e_{t}>0}\left\{\ln(e_{t})-ce_{t}^{2}-d_{t}(g_{t}-e_{t}{)}^{2}\right\}. \end{array}$$ (2)

where, *e*_*t*_ and *g*_*t*_ represent the student’s effort and aspiration levels respectively, *c* is the cost parameter associated with effort and *d*_*t*_ is a parameter capturing the psychological cost of deviating from aspirations at time *t*. The time indices indicate that the agent chooses effort repeatedly in line with the panel struc*t*ure of our data. While we take into account the fact that students need to choose effort repeatedly, we stop short of modeling this as a fully dynamic problem. This is equivalent to assuming that agents are myopic. The model remains therefore agnostic with respect to the aspiration dynamic, as the agent does not internalise the indirect effect of exerting effort on future aspirations. Our main modification to the framework by Dalton et al. [2] is to introduce the time-varying parameter *d*_*t*_, which models the differential impact of aspirations on effort in different time periods (Hence, *d*_*t*_ can be interpreted as a discount factor that adjusts the weight of aspiration discrepancies in the utility function at each time period.). There are many reasons to believe that the relationship between aspirations and effort is not stable, and might depend on how salient the outcome the agent is working towards is in their mind. Intuitively, the desire to perform well might induce more effort when the exam is next week than if it is months away.

To bring the model to the data we derive standardised measures for students effort and aspirations from our data and estimate the model parameters to match observed effort choices given aspiration levels. For more details on the estimation procedure see S4 File.

Table 3 presents the estimated model parameters. The estimated cost parameter *c* is positive and significant, indicating increasing marginal costs of effort for students and aligns with the expectation that higher effort levels entail greater personal costs. The *d*_*t*_ parameters represent the psychological cost associated with deviations from aspirations at each time period. There is a notable qualitative difference in the magnitude of the *d*_*t*_ parameters between baseline and endline on the one hand and the two follow-up surveys on the other hand. Specifically, the parameter estimates for *d*_1_ and *d*_2_ are small and very close to 0, while those for *d*_3_ and *d*_4_ are much larger and positive. Our preferred interpretation of this finding is that when the focus of students’ aspirations – the Christmas exam diet – is located in the distant future, these aspirations do not exert a strong effect on the students’ effort choices. This pattern aligns with an intuitive view of agent behavior: when aspirations are focused on a distant future, agents are less likely to prioritize them in their effort allocation. As the moment to act on these aspirations approaches, aspirations naturally become more prominent and begin to influence effort strongly.

**Table 3:** Estimated parameters and bootstrap standard errors.

Parameter	Estimate	Std. Error	95% Confidence Interval
*c*	0.558	0.012	[0.536, 0.582]
*d*_1_	0.000	0.012	[0.000, 0.047]
*d*_2_	0.015	0.041	[0.000, 0.141]
*d*_3_	0.289	0.123	[0.085, 0.598]
*d*_4_	0.196	0.100	[0.054, 0.462]

Parameter estimates obtained from the structural model outlined in equation (2). Standard errors and confidence intervals based on bootstrap with 500 replications.

## 4 Discussion and concluding remarks

Our findings indicate that the role-modelling intervention increased students’ aspirations and their stated effort in the weeks following the sessions, although the magnitude and persistence of these effects warrant cautious interpretation. These immediate responses suggest that relatively light-touch aspiration-raising activities can influence students’ motivational outlook even during periods that are typically viewed as sensitive or challenging for behavioural change. At the same time, the effects we document are short-lived: aspirations and effort converge back toward baseline levels soon after the intervention. One possible explanation is the relatively light-touch nature of our intervention, which consists of a one-off exposure to a short video. This contrasts with more immersive or sustained aspiration-building interventions – such as exposure to full-length films or multi-media narratives (e.g., [5,11,14]) – as well as interventions involving repeated contact with real-life role models in the form of alumni or teachers or parents over time ([20–22]). Relative to these designs, our results suggest that one-off interventions may be less effective at generating persistent changes in aspirations and effort.

Moreover, the rapid attenuation in the effects highlights an important insight emerging from our analysis: timing appears to play a critical role. Interventions delivered at a moment when students are psychologically responsive or when their goals feel more immediate may have disproportionately larger and more durable effects compared to similar interventions delivered earlier in the horizon.

Next, our study was conducted during the Covid-19 pandemic, a period associated with substantial disruptions to students’ academic experience and mental well-being (A growing body of evidence documents significant declines in student mental health and well-being during the pandemic ([23]).). This context may limit external validity, as responses to aspiration-building interventions could differ in more typical academic environments. In particular, the effects we document could either overstate or understate impacts in non-pandemic settings, depending on whether students were more responsive to motivational content or, conversely, less able to translate such inputs into sustained effort.

We complement our empirical analysis, by a simple structural model of effort choice estimated on our dataset. Our main contribution is to estimate a modified version of the model proposed by Dalton et al. [2], to include a time varying influence of aspirations on effort setting. Our model estimates are consistent with an “aspiration-discounting” mechanism, where students’ effort response to aspirations becomes stronger the closer the measurement is taken to the Christmas exam diet. Conversely, effort appears insensitive to aspirations early in the semester. While it seems natural to think of the effect of aspirations on effort to be mediated by some temporal distance from a goal, to the best of our knowledge we are the first to formally consider this channel and provide some quantitative evidence for it.

The existence of this channel has important implications for our own study, and the broader field. If we timed the intervention closer to the exam diet, we might have expected a larger effect on measured effort. While we cannot test this hypothesis, this points to an interesting direction for future research. More broadly the existence of this mechanism creates an important trade-off for the timing of aspiration-raising programmes, particularly in educational contexts. In these settings it is likely that effort should optimally be exerted early, as the benefits of learning compound over time. However, if aspiration discounting is substantial, then the effects of aspiration-raising interventions will be weakest at the point where the ultimate benefits from increased effort are highest. This point is particularly important if the aspiration effects of an intervention fade quickly as we found in our study. Taken together, the short-lived effects of the intervention and the suggestive evidence from the model point to an important area for future work: understanding how discounting interacts with aspirations in shaping effort. Future research exploring how to align interventions with these temporal dynamics could inform more durable and effective policies in this area.

## Supporting information

S1 File Additional tables and figures. (TEX)

S2 File Notes on the design and intervention. (TEX)

S3 File Outcome variables and measurement. (TEX)

S4 File Details on model estimation. (TEX)
